# Draft Genome Sequence of the Type Strain *Sphingopyxis witflariensis* DSM 14551

**DOI:** 10.1128/genomeA.00924-17

**Published:** 2017-09-07

**Authors:** Michal A. Kaminski, Ewa M. Furmanczyk, Andrzej Dziembowski, Adam Sobczak, Leszek Lipinski

**Affiliations:** a Institute of Biochemistry and Biophysics, Polish Academy of Sciences, Warsaw, Poland; b Institute of Genetics and Biotechnology, Faculty of Biology, University of Warsaw, Warsaw, Poland

## Abstract

Here, we present the draft genome sequence of *Sphingopyxis witflariensis* strain DSM 14551. The assembly consists of 38 contigs and contains 4,306,761 bp, with a GC content of 63.3%.

## GENOME ANNOUNCEMENT

The members of the genus *Sphingopyxis* were isolated from various environments, often contaminated with chemical compounds, such as oil or pesticides. Microorganisms from this genus showed an ability to use styrene, chlorophenols, or polycyclic aromatic hydrocarbons as the sole carbon source and have the ability to degrade them. Here, we present the draft genome sequence of *Sphingopyxis witflariensis* type strain DSM 14551, which is the only representative genome of *S. witflariensis*. It was isolated from the activated sludge of a wastewater treatment plant in Germany (1). As it was collected from a wastewater treatment plant, we were investigating which genomic features could be engaged in the survival of this bacterium in such a harsh environment.

Genomic DNA was isolated as follows: an overnight culture, grown at 23°C in a shaker at 150 rpm in Luria-Bertani medium, was centrifuged and suspended in 10 ml of 2× diluted lysis buffer (2), supplemented with 2 mg/ml lysozyme and 500 U/ml of achromopeptidase, and incubated at 37°C for 1 h. Then, 50 µl of proteinase K (40 mg/ml) was added, followed by a 30-min incubation at 37°C. The final step of cell lysis included addition of an SDS solution to a final concentration of 2%, followed by a 2-h incubation at 55°C. DNA extraction was performed using a standard phenol-chloroform protocol.

Illumina paired-end (with an average insert size of 450 bp) and Nextera mate pair libraries (with an average insert size of 8 kb) were prepared according to the manufacturer’s protocols (a KAPA HTP DNA library preparation kit for Illumina sequencing and a Nextera mate pair sample prep kit, respectively). Whole-genome sequencing of *S. witflariensis* strain DSM 14551 was performed using the Illumina MiSeq platform (2 × 300 bp) and resulted in 543,135 paired reads for the paired-end library and 1,227,060 paired reads for the mate pair library. Reads from the paired-end library were processed as follows: adapters were removed using the cutadapt script (3), and reads were filtered by length (>50) and quality (q30) (4). The mate pair reads were processed with NxTrim (5). Assembly was made using SPAdes version 3.9.1 (6). Contigs longer than 1 kb were deposited in GenBank and annotated using NCBI PGAP (7).

The genome assembly resulted in 38 contigs, containing 4,306,761 bp, with a GC content of 63.3%. The DSM 14551 genome encodes 4,166 predicted open reading frames, of which 4,038 are protein-coding genes and 61 are RNA coding genes (52 tRNAs, 6 rRNAs, 3 noncoding RNAs (ncRNAs), and 67 pseudogenes).

Detailed genome analysis showed the presence of genes encoding numerous enzymes known to be involved in the decomposition of different chemical compounds, including laccases known to oxidize a wide variety of phenolic and nonphenolic compounds; genes similar to phenylacetone monooxygenase, which has been described to be involved in oxidizing aromatic and aliphatic ketones; phthalate degradation genes (*phtABC*); and five genes annotated as dehalogenases that may be involved in halogenated compound degradation. The analyzed genome also showed the presence of copper resistance genes (*copABCD*). Moreover, the identification of conjugative transfer genes (*trbBCDEJ*) suggests that *S. witflariensis* DSM 14551 may contain plasmids in its genome.

### Accession number(s).

This whole-genome shotgun project has been deposited at DDBJ/ENA/GenBank under the accession no. NISJ00000000. The version described in this paper is the first version NISJ01000000.
